# From Intrauterine to Extrauterine Life—The Role of Endogenous and Exogenous Factors in the Regulation of the Intestinal Microbiota Community and Gut Maturation in Early Life

**DOI:** 10.3389/fnut.2021.696966

**Published:** 2021-12-17

**Authors:** Anna Socha-Banasiak, Malwina Pawłowska, Elżbieta Czkwianianc, Kateryna Pierzynowska

**Affiliations:** ^1^Department of Gastroenterology, Allergology and Pediatrics, Polish Mother's Memorial Hospital-Research Institute, Lodz, Poland; ^2^Department of Biology, Lund University, Lund, Sweden; ^3^Department of Animal Physiology, The Kielanowski Institute of Animal Nutrition and Physiology Polish Academy of Sciences, Jablonna, Poland

**Keywords:** microbiota, prenatal factors, gut maturation, early life, epigenetics

## Abstract

Differentiation of the digestive tube and formation of the gut unit as a whole, are regulated by environmental factors through epigenetic modifications which enhance cellular plasticity. The critical period of DNA imprinting lasts from conception until approximately the 1,000th day of human life. During pregnancy, besides agents that may directly promote epigenetic programming (e.g., folate, zinc, and choline supplementation), some factors (e.g., antibiotic use, dietary components) can affect the composition of the mother's microbiota, in turn affecting the fetal microbiome which interacts with the offspring's intestinal epithelial cells. According to available literature that confirms intrauterine microbial colonization, the impact of the microbiome and its metabolites on the genome seems to be key in fetal development, including functional gut maturation and the general health status of the offspring, as well as later on in life. Although the origin of the fetal microbiome is still not well-understood, the bacteria may originate from both the vagina, as the baby is born, as well as from the maternal oral cavity/gut, through the bloodstream. Moreover, the composition of the fetal gut microbiota varies depending on gestational age, which in turn possibly affects the regulation of the immune system at the barrier between mother and fetus, leading to differences in the ability of microorganisms to access and survive in the fetal environment. One of the most important local functions of the gut microbiota during the prenatal period is their exposure to foreign antigens which in turn contributes to immune system and tissue development, including fetal intestinal Innate Lymphoid Cells (ILCs). Additional factors that determine further infant microbiome development include whether the infant is born premature or at term, the method of delivery, maternal antibiotic use, and the composition of the mother's milk, among others. However, the latest findings highlight the fact that a more diverse infant gut microbiome at birth facilitates the proliferation of stem cells by microbial metabolites and accelerates infant development. This phenomenon confirms the unique role of microbiome. This review emphasizes the crucial perinatal and postnatal factors that may influence fetal and neonatal microbiota, and in turn gut maturation.

## Introduction

From the 3rd week of human embryogenesis, the digestive tube starts differentiating, leading to the formation of epithelial cells and eventually into the structural and functional gut unit (intestinal villi and crypts), along with the development of the enteric nervous system in the weeks to follow (1). The human gut is composed of the three germ layers—endoderm (which forms the epithelial lining of the lumen), mesoderm (which forms the smooth muscle layers), and ectoderm (which includes the most anterior and posterior luminal digestive structure and the enteric nervous system) (2). Besides for the well-known genetic and transcription factors which directly influence organogenesis, the early colonization of the gut with microbiota is pivotal in the development of a functional intestine and thus in the general health status of the infant, as well as that of the human in adult life. The gut microbiome provides us with both genetic and metabolic agents that we have not been required to evolve on our own, including the synthesis of various inaccessible nutrients (3). The human gut microbiome consists of a collective genome of microorganisms including bacteria, fungi, viruses, eukaryotes, and archaea and exceeds the size of all other microbial communities, with trillions of bacteria, often representing thousands of species, predominating (4). For about a century the fetus was believed to be sterile, with microbes only colonizing the newborn during birth. In the last few years however, it has been proven that the development of the structural and functional gut unit is a complex process which requires microbial input (5). Contact with the mothers' microbes and their metabolites during prenatal life may impact the infant's immune system and prepare the offspring for colonization during delivery and postnatal life (6). The mode of birth, contact between the infant and the mother's skin and breastfeeding are the next pivotal factors that determine the neonate's microbial diversity, as well as developmental and health consequences in the future (6–8). The critical period of DNA imprinting and early colonization last from conception until about the 1,000th day of human life. It should be emphasized that the gut microbiota is not only a local community of organisms, but should be considered as an active “organ” which provides the host with metabolic, trophic, and immunological benefits (9). Microbiota ensure protection against pathogens, stimulate innate, and adaptive (humoral and cellular) immune responses, regulate the development of enterocytes and take part in the synthesis of vitamins, short-chain fatty acids (SCFAs), and polyamines (9). Moreover, disturbances of the microbiome during early life are also associated with a higher incidence of chronic pediatric diseases, including obesity, allergic diseases, irritable bowel syndrome, or inflammatory bowel diseases (10).

In this review, we highlight the role of some perinatal and postnatal factors in modifying the composition of fetal and neonatal microbiota which in turn influences gut maturation processes. Moreover, we emphasize the role of the gut microbiota in the transformation of the “dead” food consumed by the host into “living integrated food,” with immunological and nutritional benefits for the host.

### Fetal Life

A research study performed in 2014, using next-generation sequencing, described a unique microbiome in the human placenta, which challenged the sterile womb paradigm (11). Results of studies that followed showed the existence of microbial communities in the fetal environment (placenta, cord blood, amniotic fluid, fetus, and meconium) and gave rise to the hypothesis of “*in utero* colonization” (12–15). The origin of this fetal microbiome is not well-known, however, the microbes may originate both from the vagina through ascendant colonization and from the maternal oral cavity/gut, through the bloodstream (5, 11, 16, 17).

The results of some previous studies performed, with an emphasis on sterile conditions, confirmed the presence of a viable mammalian fetal microbiota during *in utero* development (18, 19). Liu et al. showed that the distribution patterns of bacterial communities in the meconium of neonates is not dependent on the mode of delivery/birth, which in turn confirmed the exposure of the fetus to bacteria prior to birth (19). He et al. revealed that the microbiota in meconium shares more features with that of the amniotic fluid, compared to the maternal fecal and vaginal microbiota (20). Culture examination of meconium in 21 healthy, full-term neonates, born from healthy mothers, taken within 2 h of birth, and prior to feeding, revealed a diverse group of Gram-positive and Gram-negative bacteria with a dominance of *Enterococcus* and *Staphylococcus* (21). On the other hand, the microbiota from meconium collected from 14 preterm infants mainly consisted of *Bacillus* and other *Firmicutes* (22). Moreover, results from a study performed on human and mouse dyads demonstrate a dynamic, viable mammalian fetal microbiota during *in utero* development. Cultivatable bacteria were found in the fetal intestines only during mid-gestation, suggesting fetal exposure to viable and cultivatable bacteria during mid-gestation and subsequently to non-cultivatable bacteria during late gestation (18). These findings imply differences with regards to the ability of the microorganisms to access and persist in the fetal environment, caused by changes in immune regulation at the materno-fetal barrier during gestation, or the possibility of some of the microbes to exist in a viable, but non-cultivatable state (18, 23). On the other hand, several other previous studies have put forward arguments which contradict the possibility of infant microbial colonization *in utero* (24).

**Figure 1:**
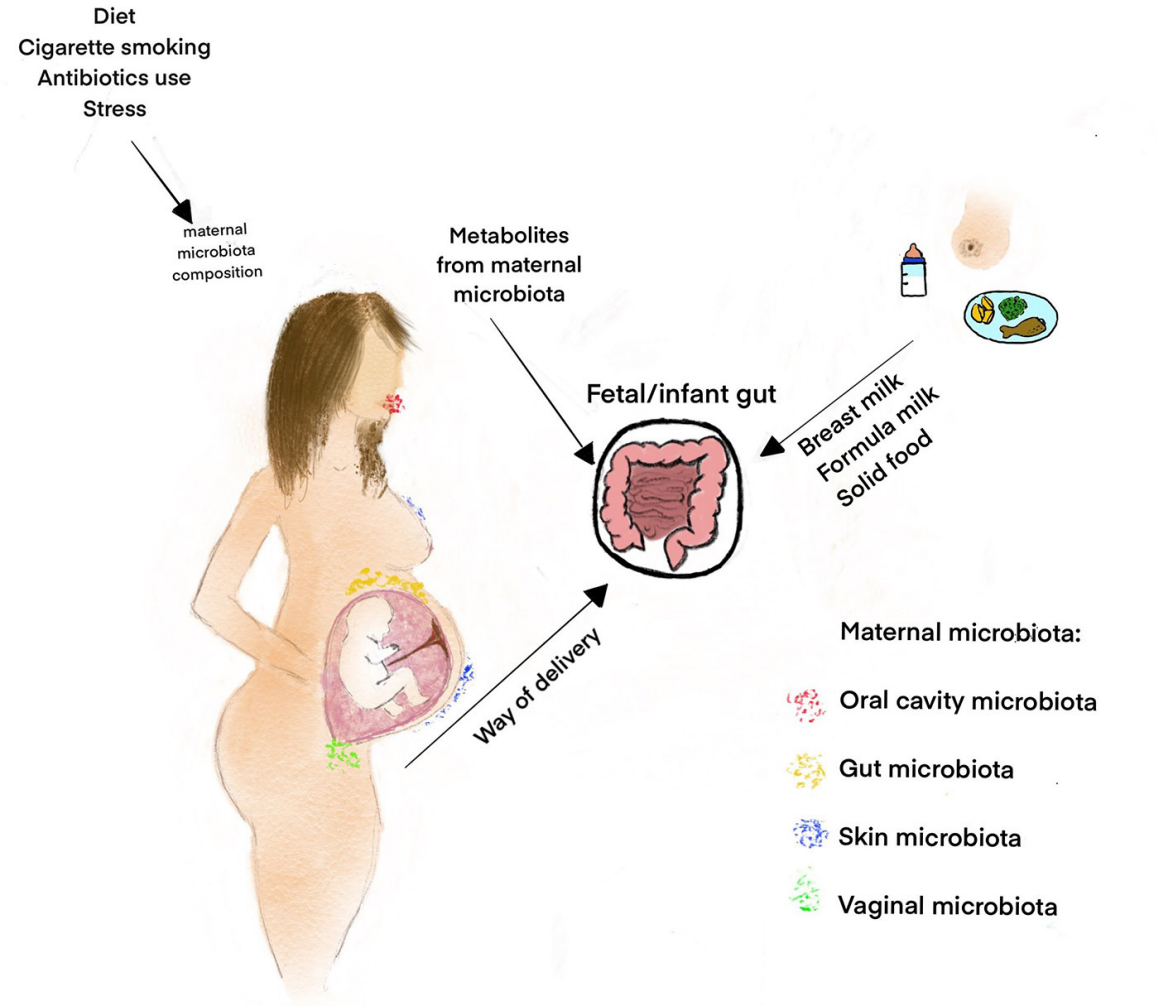
Factors influencing the gut microbiota composition in the fetal period and infancy. Some factors like maternal diet, obesity, cigarette smoking, and the use of antibiotics influence the maternal microbiota composition and, consequently, fetal microbiome (25). The fetal microbiome probably originates both from the vagina through ascendant colonization and from the maternal oral cavity/gut, through the bloodstream (5, 11). The maternal microbiota metabolites transported to the fetus through the placenta regulate fetal development, including gut maturation and prepare the offspring for colonization during delivery and postnatal life (6, 26). The other important factors that influence the early microbiota composition and diversity are the way of delivery, contact between the infant and the mother's skin and feeding introduction (27, 28).

**Figure 2:**
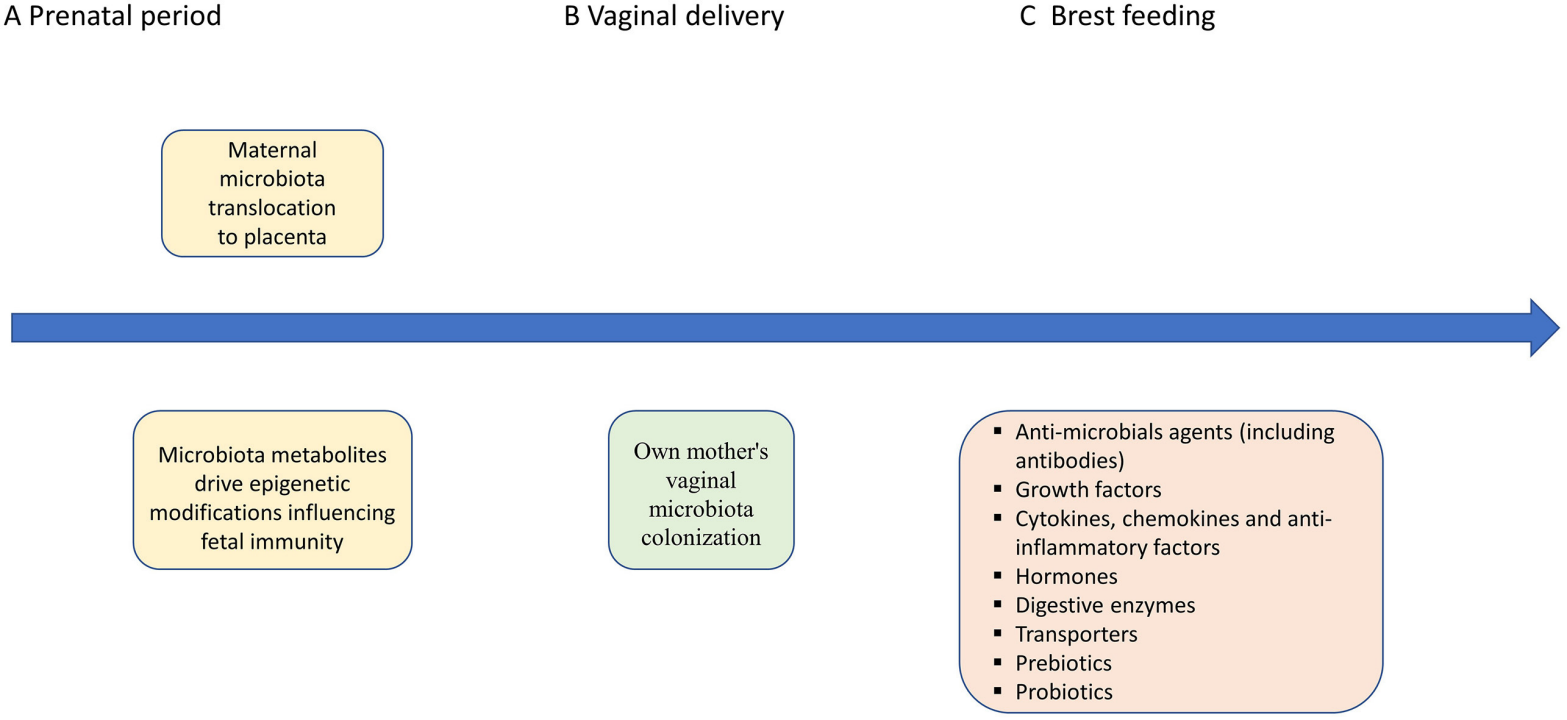
Potential mechanisms of maternal microbiota, vaginal delivery, breast feeding effects on the fetal/infant immunity. **(A)** The early microbial colonization in humans is necessary to maintain a balance between maternal and fetal immunity (30). Fetal microbiota, or their molecular signatures, may form mucosal immunity and prepare the tissues for colonization following birth (18). The maternal microbes and their metabolites have impact on immunity development by epigenetic modifications influencing Th1/Th2 balance, production of SCFAs that may induce T-cell activation and modulate IL-10 release, as well as regulation of TLRs (5, 33–35). **(B,C)** Vaginal delivery assures own mother's vaginal microbiota colonization. Breast feeding with all bioactive factors assures protective role against infections and maintains the mucosal epithelium and the development of the microbiome (36). Moreover, mother-derived active molecules (including immunoglobulins) assure gut homeostasis with regard to the newly colonizing microbiota by regulating the immune system in the GALT and shaping Innate Lymphoid Cell development in the intestine (36). Milk-derived antibodies from the mother's milk support the child with a passive immunity during the infancy (37). Short-chain fatty acids (SCFAs), Toll-like receptors (TLRs), and gut-associated lymphoid tissue (GALT).

It has previously been shown that many prenatal factors, such as maternal diet, obesity, cigarette smoking and the use of antibiotics, may influence maternal microbiota composition, and in turn that of the neonate as well (25). Besides the “still controversial” hypothesis of “*in utero* colonization,” it should be emphasized that fetal development, including gut maturation, is definitely regulated by maternal microbial metabolites, transported to the fetus through the placenta [(24, 26); Figure 1]. Molecules from symbiotic microorganisms may initiate the microbial–host mutualism *in utero*, before acquisitioning microbial biomass in the offspring. The metabolites which take part in epigenetic DNA imprinting are synthesized endogenously by the maternal microbiota or originate from food compounds that are consumed by the mother (26). On the other hand, the presence of fetal microbiota, in the absence of any infection or inflammation, supports the hypothesis that microbes colonize the fetus before birth and also play a role in the physiological development of the fetus, including that of the gastrointestinal tract (5). It was previously shown in an animal model (Cesarean-derived germfree newborn piglets) colonized with adult swine feces that resident microbiota induced the expression of genes contributing to intestinal epithelial cell turnover, mucus biosynthesis and the priming of the immune system (29). Moreover, the early microbial colonization in humans, over and above the influence of other maternal factors like hormones and cytokines, is necessary to maintain a balance between maternal and fetal immunity (30). Fetal microbiota, or their molecular signatures, may form mucosal immunity and prepare the tissues for colonization following birth (18). This is especially significant in the context of beneficial bacteria immune regulation, such as *Bifidobacteria* and *Lactobacilli* present in the human placenta (31, 32). Microbiota colonization is associated with gut-associated lymphoid tissue (GALT) development (33). Toll-like receptors (TLRs) present on the surface of various cells (macrophages, mast cells, and dendritic cells) may recognize distinct bacteria driving the development of a potential inflammatory response (5). Intrauterine bacteria have been shown to promote the development of the fetal immune system, though the TLRs (34). Moreover, the production of SCFAs that may induce T-cell activation and modulate IL-10 release is another potential mechanism by which microbes influence the immune system (5, 35). The potential mechanisms of maternal microbiota influence on fetal immunity are presented in Figure 2.

### Neonatal Period

After delivery a unique interaction or symbiotic relationship develops between the microbiome and host, which is necessary for survival. The bacterial community structure and host defenses influence the gut endothelial cells by modifying function, enhancing protection, and maintaining barrier function (38). The intestinal microbiome also participates in the synthesis of amino acids, vitamins and metabolites, which contribute to the general well-being of the host (26). Moreover, the gut microbiota also play a role with regards to the overall enzymatic function of the intestine, by ensuring the host with the necessary enzymes to digest complex carbohydrates (3). Taking into account that the human body is considered a holobiont, the genetic variation among hologenomes, including the combination of genetic content between the host and its associated microbiota, may result due to modifications in the host genome or the genomes of the constituent symbiotic microorganisms (39). These processes are essential especially for host health during the establishment of the infant gut microbiota, when trans-kingdom interactions between host and microbial cells are initiated (39).

The microbial composition of infants changes substantially in the days directly post-birth and is dependent on various factors including mode and time of delivery, maternal diet and infant diet (breast feeding vs. formula feeding), among others [(27, 36, 39, 40); Figure 1].

#### Mode of Delivery/Birth

Mode of delivery is one of the crucial factors that influence the composition of the microbiota in newborns (27). Dominguez-Bello et al. showed that infants delivered by Cesarean section (CS) harbored bacterial communities similar to those found on the skin surface, dominated by *Staphylococcus, Corynebacterium*, and *Propionibacterium* spp.; while vaginally delivered newborns acquired bacterial communities resembling their own mother's vaginal microbiota, dominated by *Lactobacillus, Prevotella*, or *Sneathia* spp. (27). Moreover, Kim et al. showed delayed establishment of intestinal microbiota in newborns delivered by CS. In the group of vaginally delivered infants the overrepresentation of starch/sucrose, amino acid, and nucleotide metabolism in gut microbiota with depleted lipopolysaccharide biosynthesis was detected as compared to newborns delivered by CS (41). Taking into account that early gut colonization affects all stages of the human development, this phenomenon may lead to the disturbance in the processes of immune maturation, metabolic programming, and in turn gut maturation in newborns delivered by CS (41, 42). The intestinal microbiota and its metabolites influence the growth and differentiation of the epithelial cells of the intestinal lumen, promote digestion and metabolism of food, take part in vitamin and amino acids synthesis and ion absorption (43). The significant differences in microbiota composition of neonates born vaginally vs. those born via CS lead to the initiation of the Vaginal seeding (VS) procedure. During VS, maternal vaginal bacteria are artificially administered to babies following birth by CS. This procedure was initiated in attempt to mimic the exposure of the offspring to the vaginal microbiota, which would normally occur during a vaginal birth (44, 45). However, the safety of the above mentioned procedure is controversial due to exposure to both vaginal commensals and potential pathogens that may lead to severe infection (44).

#### Prematurity

In the case of premature birth, post-delivery maturation of the gut is crucial for survival and the general and future well-being of the infant (46). The intestinal microbiota of preterm infants is characterized by a different biodiversity of microorganisms compared to that of full-term infants (47, 48). Moreover, available data suggests that microbiota development is driven by host biology and associated with gestational age (49). *Bacilli* initially dominate, followed by *Gammaproteobacteria*, and finally by *Clostridia* by ~37 weeks post-menstrual age (50). Prematurity predisposes the infant to gut dysbiosis with a dominance of Gram-negative bacteria of the class *Gammaproteobacteria* and its constituent families *Enterobacteriaceae, Vibrionaceae, Pseudomonadaceae*, and decreased relative abundance of *Firmicutes* and *Bacteroidetes* (51–53). Alterations in microbiota composition puts infants at risk for the development of diseases such as necrotizing enterocolitis (NEC) and chronic lung disease (51–54). Clinical observations have confirmed a link between factors produced during chronic inflammation and growth failure (55). A distinctive pattern of host microbiota interaction in a humanized gnotobiotic mouse model of intestine development has previously been described (56). In the above-mentioned studies, the pregnant mice were colonized with fecal microbiota from preterm infants with low (M_PI_-L) or high (M_PI_-H) growth rates. Microbiome analysis showed a greater contribution of *Bacteroidetes* and *Actinobacteria* in M_PI_-H-colonized mice compared to M_PI_-L mice. Moreover, it was noted that the mice offspring showed the original preterm infants' growth phenotype—M_PI_-H offspring, exhibiting a higher growth rate and advanced maturation of the intestine (well-organized tight junctions, higher goblet and Paneth cell numbers, and higher expression of intestinal epithelial maturation marker gene, Lgr5), in comparison to M_PI_-L pups. The reduced development of the M_PI_-L pups related to the overexpression of pro-inflammatory markers such as monocyte chemoattractant protein-1(MCP-1), vascular cell adhesion molecule-1 (VCAM-1) and nuclear factor kappa-light-chain-enhancer of activated B cells (NF-κB), in the intestinal mucosa (56, 57). Dougherty et al. found that the distinct microbial communities acquired by full-term and pre-term neonates generate distinct metabolomic profiles in the developing small intestine (58). Full-term infant microbial communities were characterized by a lack of diversity and an overrepresentation of various *Bacilli* spp., relative to that of pre-term infants. Therefore, the pre-term neonate metabolome was characterized by an increase in bacterially transformed products, vitamins, and amino acid derivatives that induce stem cell proliferation (58).

Despite the adequate supply of amino acids by enteral and parenteral nutrition, the gut bacteria are responsible for polyamine production (59). Putrescine is a polyamine which serves as a precursor for cellular division and increased cell proliferation (60). In the case of premature infants, ornithine and putrescine levels were found to be related to the abundance of specific bacterial groups including *Clostridia, Gammaproteobacteria*, and *Actinobacteria* (58). Moreover, the pre-term intestinal metabolome is richer in deoxycholic acid (DCA) which promote stem cell proliferation during early life (58). However, the gut dysbiosis that is usually observed in premature infants may negatively influence innate immunity. NEC has been shown to be associated with increased intestinal expression of TLR4 and decreased expression of TLR9, which is modulated by bacterial lipopolysaccharide (61). However, stimulation of this pathway is dependent on microbiota composition, since some LPS isoforms have been reported to inhibit TLR4 signaling (62). The microbiota is shaped not only by gestational age, but also by the environment in the neonatal intensive care unit, nutrition, and common clinical practices in neonatal care (46).

#### Diet

**Figure 3:**
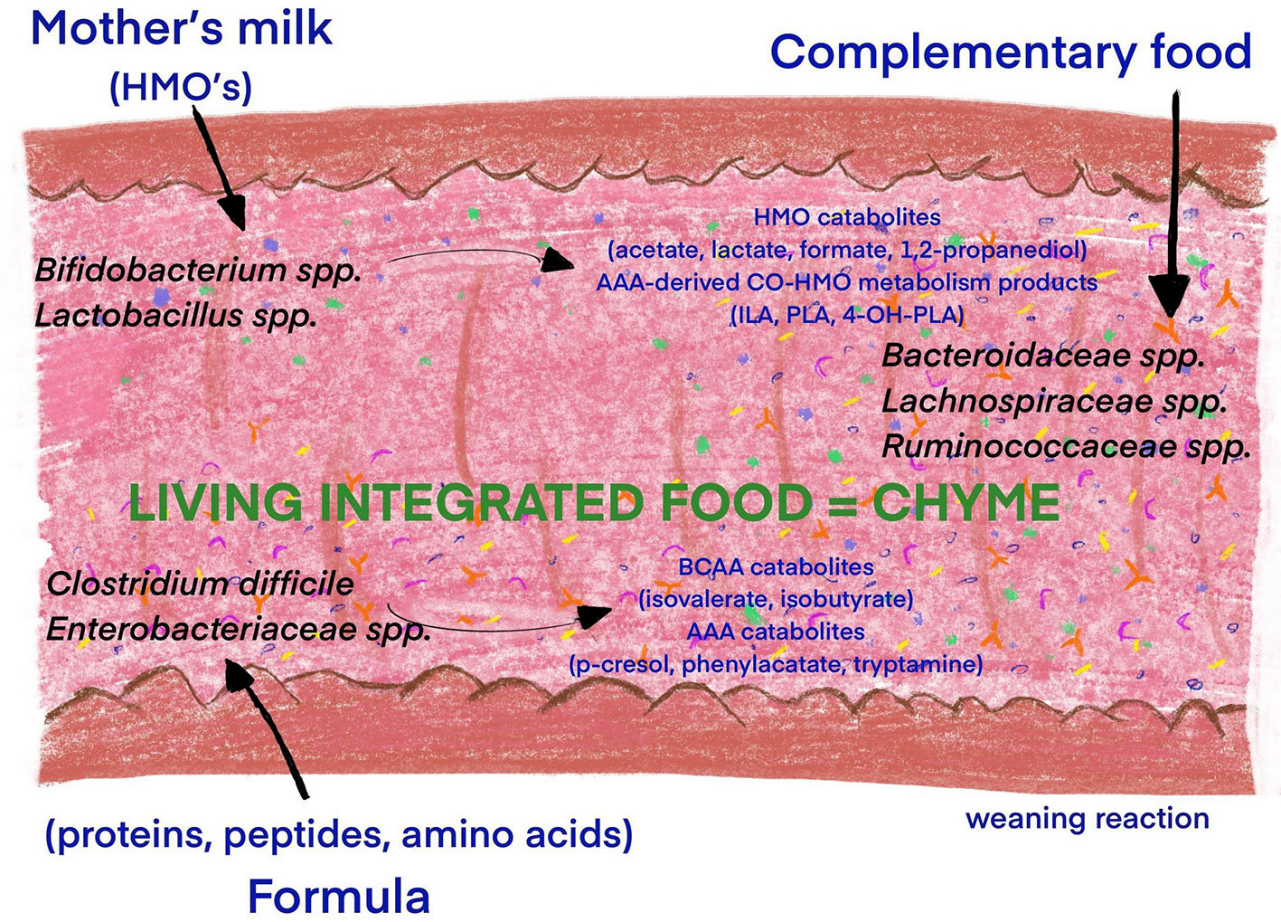
Influence of diet introduction and food composition on the infant microbiota diversity. The introduction of feeding is a milestone in the development of adaptive immunity, the production of digestive enzymes and the transformation of the consumed milk into “living integrated food” (chyme). The diet is essential in the colonization processes during the early neonatal period, as well as in the establishment of a “lifelong” microbiota composition. The microbiome of breastfed infants consists predominantly of *Bifidobacterium* (*B. breve, B. longum*, and *B. bifidum*) and *Lactobacillus* (64). The utilization of HMOs from human milk by *Bifidobacteria* is especially beneficial for the production catabolites (Acetate Lactate Formate 1,2-Propanediol) and AAA-derived Co-HMO metabolism products (65). Formula-fed infants have a more diverse microbiota, with increased abundance of *Bacteroidetes, Firmicutes, Clostridium difficile*, and *Enterobacteriaceae* and in turn BCAA and AAA catabolites (64, 65). Complementary feeding increases gut microbial diversity with dominance of *Lachnospiraceae, Ruminococcaceae, Bacteroidaceae*, and *Prevotellaceae* species and production of BCFAs and SCFAs (65, 66). Human milk oligosaccharide (HMO), aromatic amino acid (AAA), branched chain amino acid (BCAA), indolelactate (ILA), phenyllactate (PLA), 4-hydroxypheyllactate (4-OH-PLA), branched chain fatty acids (BCFAs), and short-chain fatty acids (SCFAs).

The postnatal period is crucial for continuation of microbiota colonization, as well as the development of host–microbe interactions and immune homeostasis (63). During the intrauterine period the innate immunity is highly adapted to facilitate the fetal–postnatal transition to a rapidly increasing microbial biomass and the development of long-term host microbial mutualism (26). The most important modifier of the intestinal microbiome, regardless of developmental stage, is diet (36). Gut microbiota colonization, caused by the introduction of feeding, is a milestone in the development of adaptive immunity, the production of digestive enzymes and in the transformation of the consumed milk into “living integrated food” (chyme). Exogenous factors, including the diet, are essential in the colonization of the gut by bacteria during the early neonatal period, as well as in the establishment of a “life-long” microbiota composition (Figure 3). Fulde et al. reported that enhanced expression of the flagellin receptor, TLR5, by the murine neonatal epithelium, contributes to the selection of a beneficial gut microbiota that strongly influences the composition of the microbiota throughout life (63).

##### Human Milk vs. Formula Feeding

Human breast milk is recommended as the gold standard of infant nutrition. The first few portions of mothers own milk (MOM) provide the infant with colostrum, which is low in volume but rich in immune substances, which are essential for gut development (28). The MOM portions that follow supply the infant with more nutrients, along with immunomodulatory substances including antibodies, oligosaccharides, anti-microbial agents, epidermal growth factors, hormones, digestive enzymes, and microorganisms to colonize the neonate's intestines [(28); Figure 2]. Breast milk contains a diverse population of bacteria that are transferred to the infant's gut (8). The MOM microbiome is similar with regards to composition as the skin microbiome and is dominated by *Staphylococcaceae* and *Streptococcaceae*, with lesser amounts of *Lactobacilliaceae, Corynebacteriaceae*, and other organisms (36). The microbiome of breastfed human infants consists predominantly of *Bifidobacterium* (*B. breve, B. longum*, and *B. bifidum*) and *Lactobacillus*. Formula-fed infants, on the other hand, have a more diverse microbiome (64). The pasteurized donor human milk (DHM) recommended for preterm infants when MOM is lacking, also influences the infant's metabolic phenotype and microbiota. Although preterm infants fed DHM present with microbial profiles closer to those of neonates fed MOM, compared to those fed formula, some differences were reported. Preterm infants fed MOM show a significantly greater presence of *Bifidobacteriaceae* and a lower presence of *Staphylococcaceae, Clostridiaceae*, and *Pasteurellaceae*, compared to preterm infants fed DHM (67). Piñeiro-Ramos et al. showed significant differences in the galactose, starch and sucrose metabolism pathways when comparing MOM and DHM, as well as differences in the gut microbiota composition (68). Moreover, it was shown that the protective effect of DHM is not complete probably due to destruction of the enzymes and immune molecules during pasteurization process (69, 70). MOM is the primary source of secretory IgA (sIgA) for neonates. sIgA is secreted by the B cells of the mammary gland and is shaped by the intestinal microorganisms of the mother (36). The protection provided by maternal sIgA is important to ensure a stable relationship between the microbiota and the host immune system (64). sIgA has a local gut action and provides anti-bacterial effects by binding to bacteria and preventing them from invading the mucosal epithelium (36). Beside the anti-bacterial action, sIgA may also promote physiological gut colonization with *Bacteroides* and *Firmicutes* and limit the growth of inflammatory facultative anaerobes, such as *Enterobacteriaceae*. sIgA supports the stable colonization of *Bacteroides* by acting as a carbohydrate source and through the regulation of the commensal colonizing factor (ccf), which enhances coating by sIgA and increases epithelial adherence of those bacteria (36, 71). Considering that IgG transplacental transfer takes place in the third trimester of pregnancy, immunoglobulin A, M, and G delivery through the MOM is especially pivotal in the case of premature infants, until endogenous immunoglobulin production begins (64). The action of IgG in the gut is related to the prevention of the development of potential enteric infections. The cooperation of maternal IgG with IgA ensures intestinal homeostasis, such that in the absence of either IgA or IgG, antibodies of the other isotypes are sufficient to assure immune responses. Dual recognition of microbes by both IgA and IgG assures the cooperative nature of these antibodies (72). Moreover, mother-derived IgG and IgA assure gut homeostasis with regards to the newly colonizing microbiota by regulating the immune system in the GALT and shaping Innate Lymphoid Cell development in the intestine (36).

Human milk oligosaccharides (HMOs) are another key factor in promoting favorable gut colonization assuring the protective, metabolic, and trophic roles of microbiota. HMOs consist of five monosaccharide building blocks, glucose (Glc), galactose (Gal), N-acetylglucosamine (GlcNAc), fucose (Fuc), and the sialic acid N-acetyl-neuraminic acid (Neu5Ac). HMOs are involved in the composition of neonate microbiota by regulation of colonization (73). HMOs have been suggested to inhibit the growth of pathogenic bacteria including *Streptococcus pneumoniae* and *Haemophilus influenzae* by interfering with the adhesion of the bacteria to epithelial cells (74). On the other hand, some bacteria, including *Bifidobacterium* and *Bacteroides*, metabolize HMOs for energy production (73). The utilization of HMOs by *Bifidobacteria* is especially beneficial for the production of SCFAs (36). SCFAs are the primary end-products of the fermentation of non-digestible carbohydrates available to the gut microbiota. They represent the major flow of carbon from the diet, through the microbiome, to the host (75). SCFAs including acetate, butyrate, and propionate and to a lesser degree the branched-chain fatty acids, are the main products of nutrient breakdown by microbes (76). The SCFAs may be absorbed or used as an energy source by colonocytes to maintain the colonic epithelium, however, the metabolites have other systemic effects in the host through their action as signaling molecules and involvement as regulators of gene expression. SCFAs play an important role in regulating the integrity of the epithelial barrier through the stimulation of tight junction proteins, which are essential in decreasing intestinal permeability and the translocation of bacteria and/or their cell wall components, which trigger inflammatory responses (75). The inhibition of bacterial translocation protects against the activation of TLR4, by bacterial LPS, which may in turn mediate a pro-inflammatory cascade in immune cells (75). Bridgman et al., in a study performed on infants, confirmed that breastfeeding strongly influences the composition of fecal microbial metabolites in infancy. Compared to non-breastfed infants, those exclusively breastfed were four times more likely to have a higher proportion of acetate relative to other SCFAs in their gut (77).

Formula feeding is recommended when MOM is lacking. As mentioned above, formula-fed infants have a more diverse microbiota, with increased abundance of *Bacteroidetes, Firmicutes, Clostridium difficile*, and *Enterobacteriaceae* (64). The differences in early microbiota colonization in this group of infants when compared to breast fed neonates result from higher proteins content and lack of HMOs in the formula. The formula milk usually contains excess protein, only partially digested and absorbed by upper gastrointestinal tract. Incompletely digested protein, peptides, and amino acids are metabolized in the colon by gut microbes, including opportunistic pathogenic bacteria (e.g., *Clostridium difficile, Escherichia coli*), into various metabolites such as isovalerate, isobutyrate, phenylacetate, p-cresol, and tryptamine (65).

In recent years, the infant formulas have been enriched with bioactive ingredients (e.g., probiotics, prebiotics, polyunsaturated fatty acids, synthetically produced HMOs) to mimic the composition of the human milk. However, the beneficial effects of this supplementation on both, fecal microbiome, and metabolome are discussed (65, 78–80).

##### Weaning Reaction/Complementary Food Introduction

The full maturation of the adaptive immune system is essential predominantly at weaning, when the newborn host is exposed to new antigens (26). It was previously shown that the antibodies in the milk, shaped by the mother's microbiota, are transferred to the newborn mammals during feeding and influence the development of immune system memory. Moreover, the change in microbiota composition following weaning is also pivotal for immune development. At weaning (in humans during the introduction of milk formula or solid food) the intestinal microbiota induce a vigorous immune response (“weaning reaction”) that may lead led to pathological imprinting and increased susceptibility to colitis, allergic inflammation, and cancer later in life (26). Prevention of this phenomenon relates to the action of RORγt^+^ regulatory T cells, which required bacterial and dietary metabolites—SCFAs and retinoic acid (81). Moreover, the weaning response may be also inhibited by epidermal growth factor, transferred to the infant by maternal milk (82).

The development of the human gut, including gut microbiome, from infancy to childhood is driven by many factors including complementary feeding. The World Human Organization (WHO) recommends starting complementary feeding around the age of 6 months. Late introduction of foods other than MOM may lead to the disturbances in growth and development (83). On the other hand, it was shown, that early introduction of solid foods increased the risk of childhood obesity and immune-mediated conditions (84) that might be associated with higher gut microbiota diversity and altered gut microbiota composition (85). Complementary feeding increases production of branched chain fatty acids (BCFAs) and SCFAs by microbes with potential implication for growth and development (65). Compared with HMOs, the carbohydrates from the first foods based on cereals, fruit and vegetables are highly diverse, which promotes selection of microbes (*Lachnospiraceae, Ruminococcaceae, Bacteroidaceae*, and *Prevotellaceae* species) that can utilize these complex substrates for growth (66). Moreover, Homann et al. showed that higher fiber intake and high dietary diversity were associated with the greatest impact on the gut microbiome, with stability of the gut microbiota, as solids were introduced (86). The metabolites produced by the microbiota at the onset of solid food ingestion contribute to the maturation of the gut barrier at the suckling-to-weaning transition (87).

## Conclusions

In this review, we highlight the influence of some agents on early human microbiota colonization and intestinal development. Although, according to available data, the *in-utero* colonization hypothesis is debatable, the effects of maternal microbiota metabolites on the fetus, that promote immune regulation for example, are well-documented. After delivery/birth, the unique interaction between the microbiome and host provides various benefits for the neonate including metabolic, trophic, and immunological functions. The metabolome of premature infants is characterized by increased bacterially transformed products, vitamins, and amino acid derivatives, that stimulate stem cell proliferation and gastrointestinal tract development. With regards to environmental factors, the mode of delivery/birth and breastfeeding are key factors which affect microbiota colonization and thus determine the overall health status of the organism, not only in infancy but also in adulthood.

## Author Contributions

AS-B and KP: conceptualization. AS-B: writing—original draft preparation. AS-B, KP, MP, and EC: writing—review and editing. MP: visualization. All authors have read and agreed to the published version of the manuscript.

## Funding

This study was funded by the Ministry of Science and Higher Education in Poland—subsidy for conducting research activities at the Polish Mother's Memorial Hospital—Research Institute.

## Conflict of Interest

The authors declare that the research was conducted in the absence of any commercial or financial relationships that could be construed as a potential conflict of interest.

## Publisher's Note

All claims expressed in this article are solely those of the authors and do not necessarily represent those of their affiliated organizations, or those of the publisher, the editors and the reviewers. Any product that may be evaluated in this article, or claim that may be made by its manufacturer, is not guaranteed or endorsed by the publisher.
